# Preterm Neonates Admitted to the Neonatal Intensive Care Unit of a Tertiary Care Hospital: A Descriptive Cross-sectional Study

**DOI:** 10.31729/jnma.8715

**Published:** 2024-08-31

**Authors:** Dhirendra Prasad Yadav, Vivek Kumar, Shweta Kumari Shah, Kishor Shah

**Affiliations:** 1 Department of Pediatrics, National Medical College, Birgunj, Parsa, Nepal; 2 Department of Pediatrics, All India Institute of Medical Sciences, Ansari Nagar, New Delhi, India; 3 Department of Pediatrics, Janaki Medical College, Janakpur, Dhanusha, Nepal

**Keywords:** *birth*, *neonates*, *preterm*, *prevalence*

## Abstract

**Introduction:**

Prematurity is a significant cause of neonatal morbidity and mortality, especially in low-income and middle-income countries like Nepal. However, there is a paucity of data regarding its burden. This study aimed to determine the prevalence and outcomes of preterm neonates admitted to the neonatal intensive care unit of a tertiary care hospital.

**Methods:**

This descriptive cross-sectional study was conducted among preterm neonates at a tertiary care hospital between July 15, 2022 to July 14, 2023 after obtaining ethical approval from the Institutional Review Committee (Reference number: F-NMC/557/078-079). Neonates with gestational age less than 37 weeks were included in the study. Total sampling method was used.

**Results:**

Among 980 neonates admitted to intensive care unit, preterm neonates were 112 (11.43%). A total of 69 (61.61%) neonates were outborn, and 65 (58.04%) were male. The median gestational age and birth weight were 32 weeks (interquartile range: 30-34 weeks) and 1500 gm (interquartile range: 1300-1800 gm), respectively. There were 60 (53.57%) neonates with sepsis, 51 (45.54%) with neonatal jaundice and 38 (33.93%) with respiratory distress. Death occurred in 12 (10.71%) preterm neonates in the hospital.

**Conclusions:**

The prevalence of preterm neonates was similar to other studies done in similar settings.

## INTRODUCTION

Globally, prematurity is one of the leading causes of neonatal mortality and poses significant public health issues.^1^ The World Health Organization (WHO) defines preterm birth as any delivery before 37 completed weeks of gestation or fewer than 259 days since the first day of the last menstrual cycle.^2^

Preterm neonates are at higher risk of morbidities like neurocognitive and motor impairment, malnutrition, chronic disease, and early death^1^ adding socioeconomic and psychological burden for families.^2,3^ It is necessary to conduct neonatal audits frequently due to significant geographical and temporal variations in the incidence and outcome of prematurity.^4^ Regarding preterm births, limited data are available on its prevalence and outcomes in Madhesh Province, Nepal. Survival rates for preterm neonates have increased due to interventions like corticosteroids, surfactants, and better Neonatal Intensive Care Unit (NICU) facilities. State-level estimates will update data and reflect decade-long changes.

This study aimed to find the prevalence and outcomes of preterm neonates admitted to the neonatal intensive care unit of a tertiary private hospital in Madhesh Province, Nepal.

## METHODS

This descriptive cross-sectional study was conducted at the NICU of the Department of Pediatrics, National Medical College Teaching Hospital, Birgunj, Nepal from July 15, 2022 to July 14, 2023 after obtaining ethical approval from the Institutional Review Committee (Reference number: F-NMC/557/078-079). All neonates admitted to the NICU were included in the study after written informed consent from parents. Detailed demographic details and hospital courses were recorded for neonates with a gestational age of less than 37 completed weeks. Neonates whose parents refused consent were excluded from the study. Total sampling method was used. Neonates born in the place of study were categorized as inborn whereas all deliveries that took place outside the place of study were categorized as outborn.

After enrollment, details regarding maternal age, gestational age at birth, gravida number, place and mode of delivery, antenatal check-up, use of antenatal steroids, gender, birth weight, medical problems, etc., were recorded in a predesigned performa for preterm neonates. Gestational age was calculated from the first day of the last menstrual period. If the mother was unsure of her last menstrual period, an early trimester USG scan or modified Ballard score was used to assess gestational age.^5^ Standard criteria for diagnosing the different clinical conditions were used. Sepsis was diagnosed based on clinical evidence of infection along with complete blood counts, C-reactive protein, and blood culture. Neonatal jaundice requiring phototherapy was determined by gestational age and hour-specific bilirubin level.^6,7^ Respiratory distress syndrome was diagnosed based on clinical and radiological evidence after other causes of respiratory distress were excluded. Necrotizing enterocolitis (NEC)was diagnosed based on modified Bell's staging.^7^ Hypoglycemia was defined as a blood glucose level less than 45 mg/dl.^8^ Details of hospital course and outcomes were also recorded at the time of discharge/death.

Data was entered in Microsoft Access 2007 and analyzed using STATA version 15.1.

## RESULTS

A total of 980 neonates were admitted during the study period, 112 (11.43%) cases were preterm neonates. Out of the total, there were 568 (57.95%) male and 412 (42.05%) female. The outborn neonates were 638 (65.10%) and 738 (75.30%) were delivered in hospital settings (Table 1).

Among 112 preterm neonates, 65 (58.04%) were male, and 69 (61.61%) were outborn. The median maternal age of preterm mothers was 23 years at the time of delivery (interquartile range: 21-28 years), and the median gestational age was 32 weeks (interquartile range: 30-34 weeks). The median birth weight of preterm neonates was 1500 grams (interquartile range: 1300-1800 grams) (Table 2).

**Table 1:** Baseline profile of admitted neonates and their mothers (n = 980).

Characteristics	n (%)
**Maternal age (years)**	
≤20	243 (24.79)
20-30	663 (67.66)
>30	74 (7.55)
**Mode of delivery**	
Vaginal	716 (73.06)
Cesarean	264 (26.94)
**Gender**	
Male	568 (57.95))
Female	412 (42.05)
**Place of delivery**	
Inborn	342 (34.89)
Outborn	638 (65.10)
**Birth weight (grams)**	
<2500	264 (26.94)
≥2500	716 (73.06)

**Table 2:** Baseline characteristics of preterm neonates and their mothers (n = 112).

Maternal characteristics	n (%)
**Maternal age (years)**	
≤20	27 (24.11)
21-30	72 (64.29)
>30	13 (11.61)
**Gravida**	
Primigravida	46 (41.07)
Multigravida	66 (58.93)
**Antenatal check-up**	
<4 times	33 (29.46)
≥4 times	79 (70.54)
Twin pregnancies	23 (20.54)
**Place of delivery**	
Home	5 (4.46)
PHC	1 (0.89)
Hospital	106 (94.64)
**Antenatal steroids**	
Yes	8 (7.14)
No	104 (92.86)
**Mode of delivery**	
Vaginal	55 (49.11)
Cesarean section	57 (50.89)
**Neonatal characteristics**	
**Gestational age (weeks)**	
<28	3 (2.68)
28-31	47 (41.96)
32-33	29 (25.89)
34-36	33 (29.46)
**Gender**	
Male	65 (58.G4)
Female	47 (41.96)
**Birth weight (grams)**	
<1000	7 (6.25)
1000-1499	43 (38.39)
≥1500	62 (55.36)
**Place of birth**	
Outborn	69 (61.61)
Inborn	43 (38.39)
**Age at admission to NICU**	
Day one of life	75 (66.96)
2-7 days of life	32 (28.57)
>7 days of life	5 (4.46)

Neonatal sepsis was observed in 60 (53.57%) preterm neonates (Table 3).

**Table 3:** Morbidity profile of preterm neonates (n = 112).

Morbidity	n (%)
Neonatal sepsis	6G (53.57)
Neonatal jaundice	51 (45.54)
Respiratory distress syndrome	38 (33.93)
Apnea	29 (25.89)
Birth asphyxia	18 (16.G7)
Necrotizing enterocolitis	6 (5.36)
Hypoglycemia	5 (4.46)

The median duration of hospital stay was 7.5 days (interquartile range: 4-15 days). The mortality was observed in 12 (10.71%) preterm neonates (Table 4).

**Table 4:** Outcome of preterm neonates (n = 112).

Duration of hospital stay	n (%)
≤7 days	56 (5G)
8-14 days	25 (22.32)
>14 days	31 (27.68)
**Hospital outcome**	
Discharged	82 (73.21)
Left against medical advice	18 (16.G7)
Death	12 (1G.71)

## DISCUSSION

The prevalence of preterm neonates in our study was 112 (11.43%), comparable to the prevalence seen in similar studies conducted at Nepalgunj Medical College and 12 public hospitals across Nepal (9.20-9.30%).^9,10^ A systematic review of literature on data from 107 countries revealed the global preterm births to be 10.6%.^11^ However, studies conducted within Kathmandu Valley at Tribhuvan University Teaching Hospital and Paropakar Maternity and Women's Hospital reported lower prevalence than our study (6.80-8.10%).^12,13^ Other studies conducted outside Kathmandu Valley in Nepal reported a higher incidence of preterm admission than our study (16.48-22.75%).^14-^ ^17^ The higher prevalence in these studies might be due to the differences in study hospital settings and quality of antenatal care. There is also a need for further studies on the influence of ethnicity and geographical variation in the study population.

In this study, the median maternal age was 23 years (interquartile range: 21-28 years), comparable to a similar study done at Bharatpur Hospital, Nepal (24.50 years).^17^ The percentage of primigravida mothers was 46 (41.07%), which is comparable to a previous study (42.85%)^17^ but lower than another study from Nepal (58%).^10^ In this study, 55 (49.11%) preterm neonates were born vaginally, which is similar to the study done at TUTH, Nepal (45.00%)^12^ but lower than the other studies from Nepal (61.90-66.70%).^14,17^ In our study, twin pregnancies were present in 23 (20.54%) neonates, which is higher than in other studies conducted in Nepal (5.40-13.30%).^14-17^ Multiple pregnancy increases the risk of preterm delivery due to over-distension of the uterus, which stimulates premature uterine contraction.^18^

A total of 79 (70.54%) mothers in our study had greater than or equal to four antenatal check-ups, which is higher than that of other studies conducted in Nepal (27.00-48.70%).^9,10,16,17^ Antenatal check-ups should focus on screening at-risk pregnancies, identification and treatment of infection, and dietary counseling. However, 104 (92.86%) mothers did not receive any antenatal steroids, which is higher than the study done at TUTH, Nepal (78.00%).^12^ As high proportions of our population were outborn, the reasons for not receiving antenatal steroids could not be ascertained. The possible reasons for low antenatal steroids coverage could be lack of implementation of guidelines, drug non-availability, one-third late preterm or/and late presentation to nearby hospitals. In this study, 69 (61.61%) preterm neonates were outborn, which is higher than studies done by others (15.38-45.4%).^14-17^ This is probably because our center is a tertiary care referral hospital in that region. This may also point to better perinatal and postnatal care at our center. The median gestational age of the study population was 32 weeks (range 30-34 weeks), which is similar to previous studies (33 weeks)^15^ but higher than another study from Nepal (30 weeks).^16,17^ In our study, 62 (55.36%) were moderate to late preterm neonates (32-36 weeks), which is lower than other studies from Nepal (65.30-89.50%).^10,12,14-16^

The median birth weight in this study was 1500 gm (interquartile range 1300-1800 gm) similar to studies performed from Nepal (1670-1730 gm).^15-17^ Among our study population, the majority of preterm were male 65 (58.04%), similar to other studies from Nepal (56.98-61.50%).^14,15,17^ Since most of our neonates were referred, this may indicate gender bias in favor of males seeking treatment. Further studies should explore these possibilities.

We found that sepsis was the most common morbidity seen in 60 (53.57%) neonates, which is higher than similar studies from Nepal (37.00-40.90%).^12,14^ The higher incidence may be due to most of our neonates being outborn and referred from other centers with sepsis. This indicates the importance of infection control and prevention in the treatment of preterm neonates. Few other studies have also reported a higher incidence of sepsis from Nepal (61.90-68.00%).^10,16,17^

In our study, jaundice was noticed in 51 (45.54%) neonates, similar to the studies done in Nepal (51.02-59.00%).^10,16,17^ So, immediate attention should be given to the prevention and management of jaundice in preterm neonates. Respiratory distress syndrome was present in 38 (33.93%) neonates, which is similar to studies conducted in Nepal (29.00-32.00%).^10,12^ However this finding was lower than that of other studies reported from Nepal (64.50-65.30%).^16,17^ The differences might be due to the different profiles of neonatal gestational age in both studies.

Also, apnea was noted in 29 (25.89%) neonates, which is similar to that reported by another study from Nepal (27.89%).^17^ Further, we found 18 (16.07%) neonates with birth asphyxia, which is higher than that reported from Bharatpur, Nepal (12.24%).^17^ This may be due to more number of asphyxiated cases being referred from nearby hospitals.

In our study, 6 (5.36%) neonates developed necrotising enterocolitis (NEC), which is lower than that reported in other studies reported from Nepal (13.10-14.28%).^16,17^ Again these lower NEC rates may be due to the higher median gestational age in our study. Hypoglycemia was encountered in 5 (4.46%) neonates, which is consistent with similar studies from Nepal (3.84-5.00%).^12,15^

In our study, the median duration of hospital stay was 7.5 days (range 4-15 days), similar to the other studies from Nepal (8.57-8.86 days).^14,17^ Mortality was observed in 12 (10.71%) preterm neonates, which is similar to mortality found in other studies of Nepal (11.56-12.00%)^10,12,17^ but lower in comparison to another study done in Nepal (20.60%).^16^ The favorable survival rate can be explained by improved quality care at NICU with the availability of continuous positive airway pressure, mechanical ventilation, use of surfactant, and presence of a neonatal care team round the clock. The government of Nepal has also given attention to neonatal health to achieve the Sustainable Goals by 2030 and increase the number of training for health professionals, which will help improve the quality of care of preterm neonates.

A few limitations of our study are acknowledged. Firstly, this study is single-center with a limited sample size; hence, the results may not be generalized to other settings. Secondly, the risk factors for preterm neonates could not be analyzed as many cases were referred from peripheral hospitals without proper documentation, making it difficult to collect all relevant data. Thirdly, since most of the preterm neonates were referred, some selection bias may have been introduced, potentially affecting the true prevalence.

## CONCLUSIONS

The prevalence of preterm neonates in the Neonatal Intensive Care Unit was similar to other studies conducted in similar settings; however, lower than studies conducted in different hospital settings. Further research involving multiple centers is recommended to generalize the findings and develop comprehensive strategies for reducing neonatal morbidity and mortality associated with prematurity.
